# Factors contributing to milk yield variation among cows in a cow–calf contact system in early lactation

**DOI:** 10.3168/jdsc.2021-0143

**Published:** 2021-10-22

**Authors:** Eva K. Mutua, Marie J. Haskell

**Affiliations:** 1 Royal (Dick) School of Veterinary Studies, University of Edinburgh, Easter Bush campus, Midlothian, EH25 9RG, United Kingdom; 2 SRUC (Scotland's Rural College), West Mains Road, Edinburgh, EH9 3JG, United Kingdom

## Abstract

The conventional dairy production system involves separation of the cow and calf at around 24 h after birth. Due to public concerns regarding this early separation, a few farmers are taking up the cow–calf contact system. However, some farmers have cited great variation in milk yield delivered to the parlor by cows in this sytem, with this being detrimental because it leads to low farm returns. The main objective of this study was to determine the source of variation in milk delivered to the parlor among cows in a cow–calf contact system. The study examined milk yield records for the first 28 d of lactation for 110 fall- and spring-calving cows from a farm running a full-time cow–calf contact system to investigate factors leading to variation among cows in milk delivered to the parlor. We observed great individual variation in milk yield delivered to the parlor among cows, with the range between the highest and lowest average being 22 L/d. The sex of the calf had an effect on the average milk yield delivered to the parlor: cows with female calves had higher average milk yield (Wald = 5.61, df = 1). Additionally, the lactation number of the dam affected average milk yield delivered to the parlor, with cows in their third lactation and above having the highest milk yield average (Wald = 20.90, df = 2). From the results of this study, we conclude that sex of the calf and lactation number of the dam affect milk yield delivered to the milking parlor in a cow–calf contact system.

## Highlights

• Milk yield delivered to the parlor ranged between 1 and 23 L/d in cows on a commercial farm running a cow–calf contact system.
• Cows with current female calves delivered more milk to the milking parlor than cows with current male calves.
• Milk delivered to the parlor increased with increasing lactation number.

## Graphical Abstract

**Figure:**
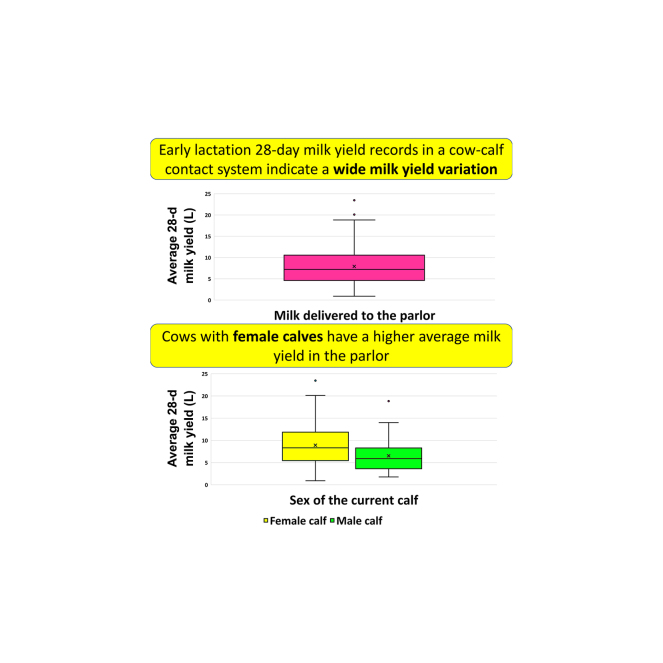
**Summary:** Cow–calf contact systems are gaining increasing attention from producers responding to the public's concerns about early calf separation. To encourage more producers to take up the system, more information is needed about how the system runs. Our study on a farm running a cow–calf contact system showed that there was a wide variation in volume of milk harvested by the milking parlor. The cows with female calves at foot had a higher milk yield in the parlor compared with cows with male calves.

Retaining cows with their calves after birth is not common practice on commercial dairy farms worldwide; rather, separation of cow and calf at around 24 h after birth is the normal practice in the conventional dairy system (Kälber and Barth, 2014). However, there is increasing public concern regarding this early cow–calf separation. Some of the publicly conveyed concerns are that early separation is unnatural, that it compromises the health of the cow and calf, and that it is considered emotionally stressful for the cow and calf (Ventura et al., 2013). The benefits of the cow–calf extended union have been documented in several studies. Cows that were suckled by their calves had a reduced rate in the occurrence of mastitis compared with cows that were only machine milked (Walsh, 1974; Krohn, 2001). Additionally, better colostral absorption (Stott et al., 1979), increased growth rate, and reduced rate of mortality are seen in suckled calves compared with artificially reared calves (Alvarez et al., 1980). Additionally, a study conducted by Wagner et al. (2012) suggested that an extended cow–calf union may lead to an enhancement of the calf's social skills.

Because of these public concerns, a few farmers are taking up the cow–calf contact system. However, farmers have concerns with the amount of milk returned to the milking parlor by the cows (Ventura et al., 2013). Studies have shown higher variation in milk yield among cows that are suckled by calves in a cow–calf contact system compared with those in a conventional milking parlor system (Bar-Peled et al., 1995; Barth, 2020). A Scottish farmer practicing the cow–calf contact system has also observed a wide variation in milk yield to the parlor by cows in his farm, with some cows giving as little as 1 L/d and some as much as 15 L/d (D. Finlay, Rainton Farm, Dumfries and Galloway, Scotland; personal communication).

Both dam and calf factors can affect milk yield in the cow–calf contact system, leading to variation in milk yield between cows. It is well known that cow age or lactation number affects milk production (Ray et al., 1992). Milk yield also varies with cow breed (ICAR, 2021). There is also some evidence that sex of the calf may affect “investment” in that calf. Studies in other species have shown that maternal investment favors the sex of offspring that is most likely to increase the reproductive success of the mother (Trivers, 1972). Thus, the observed variation in milk yield between cows in a cow–calf contact system could be due to the sex of the calf, reflecting a sex-biased maternal investment pattern (Trivers, 1972). An alternative hypothesis is that the greater birth weight and growth potential of male calves results in a difference in milk intake (Festa-Bianchet et al., 1996; Landete-Castillejos et al., 2005). As milk let-down is mediated by oxytocin, factors affecting oxytocin release during the machine milking procedure may result in variation in milk delivered in the parlor. Oxytocin is known to be affected by stress (Bruckmaier et al., 1993), so any degree of fear or anxiety experienced by individual cows in the parlor may result in some inhibition of oxytocin release, and thus affect milk delivery. Additionally, in a study comparing machine-milked cows with cows nursing calves and also being machine-milked, de Passillé et al. (2008) observed that nursing cows had lower oxytocin levels during milking, resulting in lower milk ejection. Thus, the method of milk harvesting (a calf or a milking machine) and the associated oxytocin response may be another source of variation in the milk delivered to the parlor.

This study aimed to investigate both maternal and calf factors potentially affecting milk delivered to the parlor to be able to understand milk yield variation among cows in cow–calf contact systems. The study was conducted on a commercial farm running a cow–calf contact system, which allowed us to evaluate the hypotheses that calf sex, dam parity, dam breed, sire breed, and seasonal calving group of the dam contributed to milk yield variation among cows in a cow–calf contact system.

The study was conducted after ethical approval from The Royal (Dick) School of Veterinary Sciences (R(D)SVS), Veterinary Ethical Review Committee (VERC; reference number 63.20). It was carried out at Rainton Farm in Dumfries and Galloway, Scotland. The farm is an organic dairy farm that runs a cow–calf contact system. The cows and calves are managed together with full contact for the first 6 wk of life and then are separated at night only from this age on. Weaning and full separation occur by 5 to 6 mo of age. The farm had a total of 120 cows, including Montbéliarde, Holstein, Ayrshire, and Swedish Red crossbreeds. Aberdeen Angus and Holstein Friesian bulls were used to sire the calves of these cows during the period of this study.

The cows and calves were all housed in an indoor barn in winter and fall but let out to pasture in late spring and during summer. There were 2 lactating groups in the herd: spring-calving and fall-calving cows. The 2 calving groups were housed separately within the barn. Freestalls with mattresses covered with sawdust were provided for the cows. The freestalls were separated by flexible plastic piping. Additionally, the barn had sections that housed dry cows and a calving section with calving pens where cows calved and were housed with their calves for the first 3 d after calving. There was a tandem milking parlor situated within the barn where the cows were milked.

The cows were fed fresh silage every other day with the aim of a clean feed barrier in the morning before feeding. Additionally, the silage was pushed up a minimum of 4 times a day. Concentrate was added on top of the silage every night at a rate of 1 kg/cow. The cows were given additional organic concentrate feed in the milking parlor. As the total milk yield is unknown in a cow–calf system, the amount fed in the parlor was based on an estimation of yield from a standard parlor and took into account the stage of lactation.

Each cow's first milking was made 24 h after calving, and cows were then milked once a day at 0800 h. During milking, the cows were gently separated from the calves and led to the milking parlor.

The study used milk yield records from the farm for 2019/2020. Data on milk yield were recorded in the milking parlor, stored in the farm management software (DairyPlan, GEA), and downloaded from the software for use in this study. The data set collected included milk yield records for each cow, from the day of the first milking until d 28 after entry to the milking herd. The first milking took place at the first morning milking but at least 24 h after calving. The following cow- and farm-level factors were assessed in the analysis: lactation number, cow breed, sex of calf at calving, breed of the sire of the calf (Aberdeen Angus or Holstein Friesian), and calving group (spring and fall). Cows with twins (n = 4) were removed from the data set. Two calves died within the study period, so data from their dams were removed. The average milk yield was calculated from the records for the first 28 d of lactation for each cow. Cows were grouped into 3 classes, with class 1 being cows in their first lactation (n = 37), class 2 being cows in their second lactation (n = 26), and class 3 being cows in their third lactation and above (n = 41). The cow breeds were Montbéliarde (n = 53), Holstein-Friesian (n = 34), Swedish Red (n = 9), and Ayrshire (n = 6); breed was unknown for 2 cows. Forty-six of the cows had male calves and 58 had female calves. There were 60 cows in the fall-calving group and 44 in the spring-calving group. Health treatment records were checked. No cows were treated during the study period, so no exclusions were made due to health issues. No power calculations were carried out because this was an observational study that included all animals calving during the project period.

The data were analyzed using a generalized linear model in Genstat (19th ed., VSN International). Histograms were used to confirm that milk yield followed a normal distribution. The treatment factors lactation class, sex of calf, cow breed, calf sire breed, and calving group (spring vs fall calving) were fitted as the maximal model, with the 28-milk yield average fitted as the response variate.

There was considerable variation between cows, with the highest average milk yield among the cows being 23 L/d and the lowest being 1 L/d, with an average of 8 L/d. There was no effect of cow breed (Wald = 6.46, df = 4,91; *P* = 0.177), breed of the calf's sire (Wald = 1.22, df = 4,91; *P* = 0.873) or calving group (Wald = 0.20, df = 1,91; *P* = 0.660) on milk yield average, so these factors were removed from the final model.

There was an effect of lactation class on the 28-d milk yield average (Wald = 25.52, df = 2,100; *P* ≤ 0.001), with cows in their third lactation and above having the highest milk yield average (9.8 ± 0.74 L) and cows in their first lactation having the lowest milk yield average (5.2 ± 0.51 L), as shown in Figure 1. There was an effect of calf sex on the 28-d milk yield average (Wald = 9.94, df = 1,100; *P* = 0.002), with female calving cows having higher milk yield average, as shown in Figure 2.

**Figure 1:**
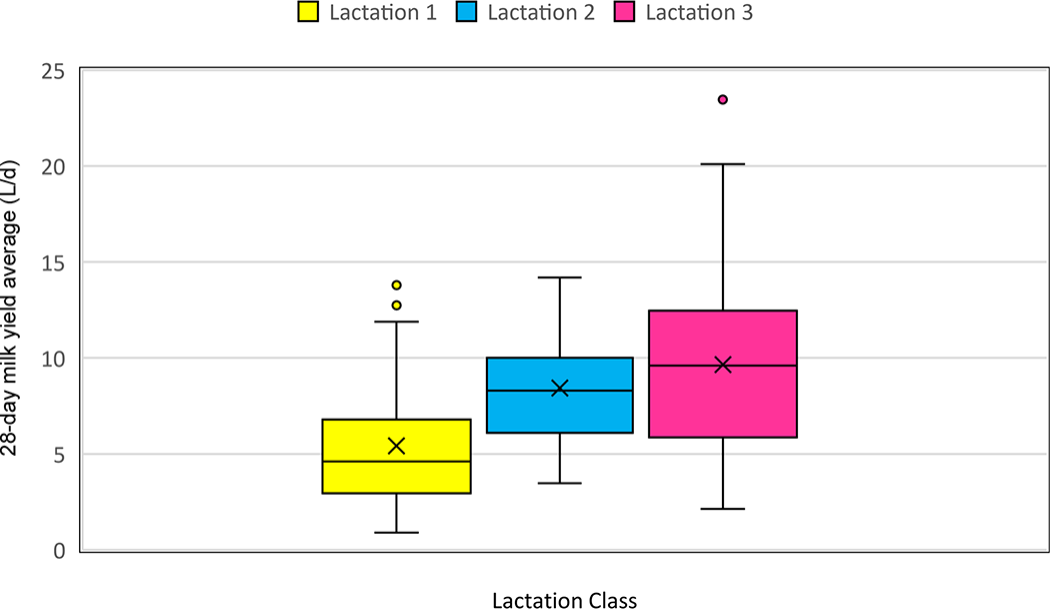
Boxplots showing milk yield (L/d) in the parlor for the first 28 DIM for dairy cows of different lactation classes nursing their calf [n = 37 (lactation 1); n = 26 (lactation 2); n = 41 (lactation 3)]. The upper and lower boundaries of the box represent the 75th and 25th percentiles; the midline represents the median; and × represents the mean. The whiskers show the range from minimum to maximum values excepting any outliers, which are represented by circles.

**Figure 2:**
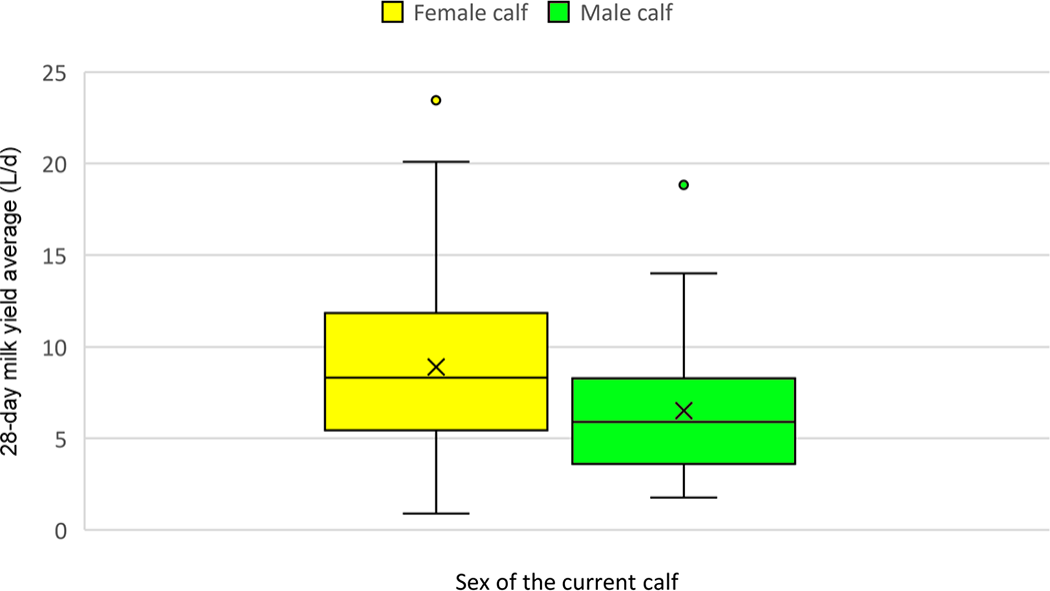
Milk yield (L/d) in the parlor for the first 28 DIM for dairy cows nursing their calf separated by sex of offspring (n = 58 cows with female calves and n = 46 cows with male calves). The upper and lower boundaries of the box represent the 75th and 25th percentiles; the midline represents the median; and × represents the mean. The whiskers show the range from minimum to maximum values excepting any outliers, which are represented by circles.

The study found high levels of milk yield variation among cows in this cow–calf contact system. Previous studies using a cow–calf contact system have also found high variation in milk yield in cow–calf contact systems (Bar-Peled et al., 1995; Barth, 2020).

Three hypotheses can explain the difference in milk harvested according to the sex of the calf. The first is that the difference in mean yield is due to higher demand by male calves, which have higher birthweights and greater growth potential (Festa-Bianchet et al., 1996; Landete-Castillejos et al., 2005). Additionally, a study by Wolff (1988) suggested that in some sexually dimorphic ungulates, male offspring suckle longer than females, and additional suckling and milk intake is likely needed to support this higher growth in male calves. The second hypothesis is that this is an example of sex-biased investment meant to maximize the reproductive success of the dam and offspring (Trivers, 1972). Previous studies on variation in maternal care during the early development of offspring have cited a sex bias favoring male offspring in ungulates (Wolff, 1988; Birgersson et al., 1998). For instance, a study by Landete-Castillejos et al. (2005) showed that Iberian deer dams with male offspring produced milk with a higher protein content, an observation that supports the sex bias toward male offspring. The third hypothesis that could support the results showing a difference in milk yield based on the sex of the calf is an effect of prenatal fetal programming in milk production. A study conducted by Hinde et al. (2014) showed that Holstein cows had biased milk production programmed during pregnancy as a function of fetal sex, with higher milk production shown in cows that carried a female fetus. However, a study conducted in Danish cattle showed that cows produced more milk when they carried a male fetus (Græsbøll et al., 2015). We were not able to distinguish between these hypotheses in this study. Due to this disparity of observations, the most parsimonious explanation of the results of this study leans toward the first hypothesis, which states that male calves with a higher growth rate and greater demand (Festa-Bianchet et al., 1996) suckled a higher amount of the produced milk, resulting in lower delivery to the milking parlor by the cows.

The results of this study aim to help farmers understand the variation in milk yield between cows in a cow–calf contact system, which may be a barrier to adoption of the system. The results of this study indicate that the various dam and calf factors affecting maternal investment affect the ultimate delivery of milk yield to the milking parlor. However, several management interventions can be made in cow–calf systems that could reduce milk yield variation among the cows. This may include using sexed semen to produce more female offspring or increasing the amount of feed given to cows with male calves, which may increase milk yield.

In this study, we were not able to factor in calf birth weight and growth rate as variables affecting milk yield delivered to the parlor because the farm is a commercial farm and did not weigh the calves. The factor of twin calving, which is a possible cause of variation in milk delivered to the parlor, was not evaluated because of the small numbers of cows with twin calves on the farm. Both factors may affect cow total milk yield and variation in milk delivered to the parlor and should be investigated further. Another potential factor affecting variation in milk yield in cows is the level of oxytocin release. As mentioned above, the ejection of alveolar milk depends on the release of oxytocin during milking (Bruckmaier et al., 1993). A study by de Passillé et al. (2008) found that oxytocin release was markedly higher during machine milking for cows always milked by machine compared with cows that both nursed their calves and were milked by machine. Lower oxytocin levels may result in less milk being delivered to the parlor by cows nursing their calves, but as stress also affects the release of oxytocin (Bruckmaier et al., 1993), differences between cows in their fear responses to parlor milking may affect milk let-down and be another source of between-cow variation in yield. Intensive blood sampling procedures would be required to assess the variation between cows in their oxytocin release during parlor milking in a cow–calf contact system, but such an experiment would further our understanding of milk production in this system.
